# The choice is yours: Binding and retrieval of free-choice responses

**DOI:** 10.3758/s13414-025-03168-6

**Published:** 2026-02-23

**Authors:** Maria Nemeth, Christian Frings, Birte Moeller

**Affiliations:** 1 Department of Psychology, Trier University, D-54286 Trier, Germany; 2 Institute for Cognitive Affective Neuroscience (ICAN), Trier University, Trier, Germany

**Keywords:** Action control, Response–response binding, Free-choice, Retrieval, Event file

## Abstract

Feature binding has emerged as an important mechanism in the coordination of human action control. Apparently, the simple mechanisms of binding of individual features and retrieval of recently integrated episodes play a significant role in the selection, initiation, and execution of goal-directed actions. Importantly, ideomotor approaches suggest that the stimulus-driven (i.e., forced-choice) as well as the internally generated (i.e., free-choice) activation of a response representation should lead to facilitation of that response in behavior. Against these predictions we separately investigated binding and retrieval of free-choice response in modified response–response binding tasks. In Experiment 1a, we investigated whether binding and retrieval of responses influence a free-choice action and found that response–response binding effects influenced performance in a current free-choice action. In Experiment 1b, we investigated whether a free-choice response can initiate the process of event file retrieval and found response–response binding effects, indicating that event file retrieval can be triggered by a free-choice response. In Experiment 1c, we investigated whether a free-choice response can be bound to another response and found response–response binding effects, indicating shared representations of free-choice and forced-choice responses. These findings are in line with ideomotor approaches suggesting that the same mechanisms of binding and retrieval also operate on representations of free-choice actions. They further demonstrate that both processes can dynamically operate across extended action sequences that integrate both forced-choice and free-choice actions, thereby highlighting their general role in flexible action control. Data of the experiments are available at OSF (https://osf.io/t7pjb/?view_only=1af9b6ce0c1c4e19ab7a79a4b0bd176f), and none of the experiments was preregistered.

Performing actions requires selecting an appropriate action from a range of alternatives, a process often considered a hallmark of voluntary action (Rae et al., 2014). Action selection can occur exogenously, when actions are triggered in response to external stimuli in the environment—for example, when hitting the brake when the traffic lights turn red. However, humans also frequently engage in endogenous decision-making, choosing for themselves how and when to act—for example, when walking in the kitchen to make a cup of coffee. Since actions are embedded in a continuous stream of ongoing behavior (e.g., Prinz, 2009; Stränger & Hommel, 1996; see also Herwig, 2015), the ability to have control over actions on the basis of both external cues and internal plans, especially in longer sequences of action, is fundamental to daily life (Obhi et al., 2009).

A central debate in the action control literature concerns whether *free-choice actions* (endogenously guided, arbitrary selections among equally plausible alternatives; Berlyne, 1957) and *forced-choice actions* (exogenously guided selections between specified alternatives; Obhi et al., 2009) are governed by distinct cognitive mechanisms. According to ideomotor theory, humans are able to perform actions by anticipating their desired sensory consequences (James, 1890; see Shin et al., 2010, for reviews). Since then, numerous studies have provided support for the assumption that the representation of an intended action effect is not only used to select a certain action, but is the cognitive process in planning and initiating actions (Kunde et al., 2004; Pfister, 2019; Pfister & Kunde, 2013). For instance, when actions are contingently followed by particular effects (e.g., specific tones), participants are subsequently faster in initiating and more likely to choose learning-compatible actions over incompatible ones upon the presentation of effects (e.g., Elsner & Hommel, 2001). However, subsequent work suggested that this effect may be limited to free-choice actions: when comparing free-choice and forced-choice tasks, action-effect learning was only observed for free-choice actions, leading to the conclusion that ideomotor action control may be restricted to endogenously generated actions only (Herwig et al., 2007). Supporting this view, several authors have proposed that endogenous and exogenous action selection rely on separable cognitive mechanisms and distinct neurophysiological systems (e.g., Herwig & Waszak, 2009; Jahanshahi et al., 1995; Obhi & Haggard, 2004; Rowe et al., 2010; Waszak et al., 2005; Wiese et al., 2004). In contrast, more recent findings suggest no qualitatively separable cognitive mechanisms in the selection and control of forced- and free-choice actions (e.g., Janczyk et al., 2017, Janczyk, Nolden et al., 2015b; Pfister & Kunde, 2013). Yet surprisingly little research has directly compared the two action types, particularly in situations requiring flexible coordination of longer action sequences.

Event-coding accounts in ideomotor context offer a valuable framework for addressing this question. According to the theory of event coding (TEC; Hommel et al., 2001), features of a (planned) action and experienced features in the environment are bound and stored in a common feature compound representing characteristics of the action episode, a so-called event file (Frings et al., 2024; Hommel, 1998, 2004). Reencountering individual features of an event file in subsequent episodes can automatically retrieve previously bound features, including the previous action. This memory-based retrieval of previously formed action plans provides an efficient short-cut for current performance: it facilitates performance of the retrieved response in a current episode but hampers performance of responses different from the one retrieved (i.e., partial repetition costs). Crucially, the binding and retrieval in action control [BRAC] framework (Frings et al., 2020) further specifies binding and retrieval as independent and separable processes.

Event coding approaches such as TEC and BRAC stress that the intentional (i.e., free-choice) or unintentional (i.e., forced-choice) reactivation of response-effect representations leads to facilitation of associated responses in behavior (Hommel, 2013). That is, although ideomotor approaches as BRAC inherently assume that spontaneous feature binding processes and retrieval of event files should occur for both forced and freely chosen actions, this has not yet been exhaustively investigated. To date, the prevailing body of evidence supports binding and retrieval processes *influencing free-choice actions* (e.g., the presentation of an action effect automatically activates the representation of the recently integrated action influencing free-choice performance; for recent perspectives, see Hoffmann et al., 2007; Kunde et al., 2007). Interestingly, a study by Richardson et al. (2020) demonstrated partial repetition costs for free-choice actions that were planned but not (yet) executed in an action planning task. These findings suggest similar representations of forced-choice responses and free-choice responses and suggest that free-choice actions themselves can be integrated into event representations alike forced-choice actions.

In consequence, if we assume that the same mechanisms of binding and retrieval apply to both forced-choice and free-choice actions as suggested (e.g., Hommel, 2007; Moeller et al., 2019; Richardson et al., 2020), we should then expect that not only would prior forced-choice actions affect current free-choice actions, but also that previous free-choice actions would reciprocally influence current forced-choice actions. However, if this is not the case, the explanatory scope of BRAC would be substantially reduced as binding and retrieval would not constitute fundamental mechanisms for the planning and initiation of actions across different types of action selection. Rather, their role in everyday behavior would be more constrained than BRAC postulates, being limited to action contexts in which forced-choice structures dominate. In other words, action control via binding and retrieval would only be able to operate as long as all actions within an action sequence share the same mode of action selection.

To the best of our knowledge, no studies have yet investigated free-choice actions with a focus on differentiating between binding and retrieval of free-choice actions. That is, if a free-choice response is not only influenced by previous action episodes but is able to initiate binding and retrieval processes itself. In fact, no studies have explored action control in free-choice scenarios that involve more complex actions, where individual actions are embedded in longer action sequences and require choices among multiple action alternatives. That is, in the mentioned studies above investigating free-choice responses, a first (prime) event typically preceded a second (probe) event, in which participants performed a free-choice response: either by repeating the prime response or by performing the alternative response. Thus, the free choice consisted of choosing between two action alternatives. In the face of such prime–probe episodes, the processes underlying the observed stimulus-response binding effects are assumed to reflect processes central to most simple actions (Frings et al., 2007, 2020). However, binding is not restricted to individual episodes, but is found to form higher-order representations in connecting separate action episodes, as found in binding between individually planned and executed responses, termed response–response binding (Moeller & Frings, 2019). The response–response binding paradigm therefore provides an ideal approach for a closer investigation of how binding and retrieval mechanisms extend beyond simple actions to influence the coordination of action sequences involving free-choice responses.

In the response–response binding task (Moeller & Frings, 2019), participants perform a sequence of four individually planned and executed responses. That is, two prime responses (R1 and R2), which can either repeat or change relative to their corresponding probe responses (R1 and R2), however, can never repeat within prime or probe. The central assumption is that the planning and execution of two prime responses leads to the binding of their response features and their integration into the same event file (see Nemeth et al., 2024, for the role of response execution for response–response binding). In a subsequent probe, repeating one of these responses as probe R1 is theorized to trigger the retrieval of the previously bound response. If the retrieved response now matches the currently required probe response R2, performance is facilitated (e.g., faster reaction times and lower error rates) and if they mismatch, performance is impaired (e.g., slower reaction times and higher error rates). These combined benefits and partial repetition costs of retrieving matching or mismatching responses are referred to as response–response binding effects and are considered as evidence of the successful retrieval of previously created response–response bindings. Importantly for the present study, the paradigm allows us to distinguish between the two processes by assuming that binding occurs during the planning and execution of prime responses, whereas retrieval is initiated when a prime response is repeated in probe (R1).

## The present study

To investigate response binding and retrieval in a free-choice scenario, we conducted three experiments. In all experiments, we used a modified response–response binding paradigm. In Experiment 1a, we investigated whether binding and retrieval of responses influence a current free-choice action. For this, we measured binding and retrieval of responses when probe response R2 was freely chosen. In Experiment 1b, we investigated whether a free-choice response can initiate the process of event file retrieval. For this, we measured binding and retrieval of responses when probe response R1 was freely chosen. Finally, in Experiment 1c, we investigated whether a free-choice response can be bound to another response. For this, we measured binding and retrieval of responses when prime response R2 was freely chosen.

In free-choice tasks, binding can be measured via response time (RT) and error rate differences, or by the facilitation of response repetitions (for both see Herwig & Waszak, 2012; Moeller et al., 2019). When using RT and error rates as the dependent variables, binding is measured as the influence of previous events on the reaction time and error rate of performing a current action. In contrast, previous studies on stimulus-response bindings in free-choice used response repetitions as the dependent variable of interest to measure how repeating a stimulus, rather than changing it, facilitates response repetitions (compared to response changes). In these studies, larger response repetition rates for stimulus repetition compared to stimulus changes indicated binding.

To account for both possibilities, in Experiment 1a we measured response–response binding effects in RTs and additionally measured the retrieval of response–response bindings influencing response repetition rates. That is, the facilitation of R2 response repetitions (compared with response changes) when the R1 response repeats rather than changes. In Experiment 1b and Experiment 1c, we measured response–response binding effects in RTs and error rates^1^.

**Fig. 1:**
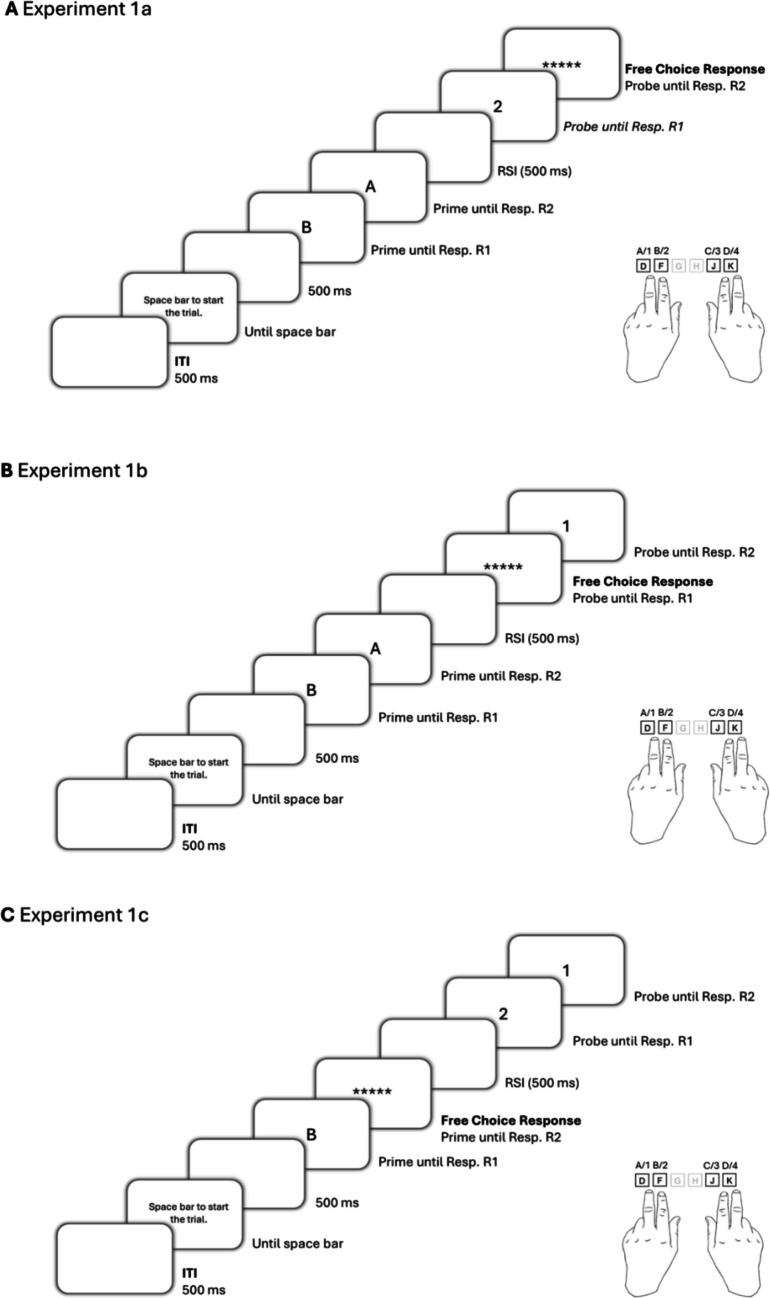
Sequence of events in one trial in Experiment 1a, Experiment 1b, and Experiment 1c. *Note*. Participants responded with their index and middle fingers of both of their hands to the identity of individually presented digits and letters. Five asterisks indicated that this response should randomly be given in free-choice. A new trial was started by pressing the space bar. Stimuli are not drawn to scale. RSI = response–stimulus interval

We expected that if a free-choice response suffices for the activation of the corresponding response representation, response–response binding effects should not only influence performance in a current freely chosen action, but a free-choice response should be able to trigger event file retrieval as well as to be bound to another response. To anticipate the results, we found response–response binding effects emerging if the current response (Experiment 1a), the retrieving response (Experiment 1b), or the to-be-bound response (Experiment 1c) was made in free-choice.

## Experiment 1a

### Method

#### Participants

Previous studies investigating response–response binding effects, typically reported medium to large-sized effects (e.g., Moeller & Frings, 2019: ${{\upeta }_{P}}^{2}$ = .29 and ${{\upeta }_{P}}^{2}$ = .42). Thus, we were interested in at least a medium sized response–response binding effect. Seventy-three participants of Trier University participated in the experiment. Following previous studies and to investigate binding and retrieval influences in free-choice (e.g., Hommel, 2009; Moeller et al., 2019) we excluded 19 participants because they repeated the prime response R2 as probe response R2 in more than 90% or less than 10% of the trials (for the impact of strategy use in free-choice designs, see Naefgen et al., 2018; Vogel et al., 2018; Weller et al., 2017). The final sample consisted of 54 participants (40 women, 13 men, one other, 47 right-handers). According to a power analysis with the program G*Power (Faul et al., 2007), this size was sufficient to find a basic response–response binding effect (statistically as the two-way interaction of the factors R1 relation and R2 relation; Moeller & Frings, 2019) of ${{\upeta }_{P}}^{2}$ = 0.20, with alpha = .05 (two tailed) and a power of 1 − ß = .95. The median age was 22 years (range: 18–34 years).

Participants were recruited via Trier University’s participant platform (Sona Systems; sona-systems.com) and performed the experiment online on the experimental platform Pavlovia (Peirce & MacAskill, 2018). All participants consented via online form before participating and received course credit as compensation. This study was done in accordance with the ethics guidelines declared by the ethics committee of Trier University. The ethics committee of Trier University declared all simple behavioral studies in accordance with their guidelines exempt from any further examinations by the committee.

#### Design

The design comprised two within-subjects factors, namely response R1 relation from prime to probe (response repetition vs. response change) and response R2 relation from prime to probe (response repetition vs. response change).

### Apparatus and stimuli

The experiment was programmed in PsychoPy (Peirce et al., 2019; Version 2023.2.3) and ran online via Pavlovia (Peirce & MacAskill, 2018). Instructions and stimuli were shown in white (Font: Arial; Font size 35 pixels) on a grey background (RGB_255_: 0, 0, 0). Stimuli were the digits 1, 2, 3, and 4 and the letters A, B, C, and D, as well as five asterisks (*****) indicating a free-choice response. Participants responded by pressing one of four keys (D, F, J, or K) on the computer keyboard.

#### Procedure

Instructions were presented at the center of the screen. Participants were instructed to place their middle and index fingers of both hands on the keys D, F, J, and K. Their task was always to classify each individually presented letter or number by pressing the corresponding key: using their left middle finger for the letter A and the number 1, their left index finger for B and 2, their right index finger for C and 3, and their left middle finger for D and 4. Participants were encouraged to respond as quickly as possible while also ensuring a high level of accuracy. For free-choice responses, participants were instructed to select one of the four response keys randomly but were strongly encouraged to not always use the same key throughout the experiment: “Sometimes you will also see these asterisks *****. Here, you can decide on your own which of the four keys (D, F, J, K) you want to press. Please do not always press the same key for these asterisks throughout the experiment but simply try to press the key that feels right at that moment. Do not follow any particular strategy here but follow your gut feeling!”.^2^

A training consisting of 16 trials was completed before the main experiment. In training trials, participants received performance-contingent feedback after both prime and both probe training displays (for a correct response: “correct”; for a wrong response: “WRONG!”; translated from German). In the experimental block, participants only received error feedback immediately following the erroneous response.

The experimental block consisted of 400 trials. In each trial, participants had to execute four responses: Two prime responses (R1 and R2) in forced-choice, a probe (R1) response in forced-choice, and finally a probe (R2) response in free-choice. Responses could either repeat or change from prime to probe (only from prime R1 to probe R1, or prime R2 to probe R2). The factor relation of response R1 between prime and probe (repetition vs. change) was varied orthogonally, resulting in 200 of each of the two conditions R1 repetition and R1 change. The response R2 relation from prime to probe was determined by the free-choice of probe response R2 (repetition vs. change).

A single trial consisted of the following display sequence: An instruction was presented indicating to press the space bar (see Fig. 1). A blank space appeared for 500 ms and was followed by the first prime digit or letter, indicating prime response R1, until the participant pressed one of the four response keys. Then the second prime stimulus appeared, indicating prime response R2, until a response was detected, which was followed by a blank space for 500 ms (response–stimulus interval). Then the first probe stimulus appeared, indicating probe R1 until a response was detected. Probe R1 was then always followed by the five asterisks, indicating a random key should be pressed in free choice. Response detection of probe R2 ended the trial. Between the trials, a blank screen appeared for 500 ms (intertrial interval), before the instruction indicated that the next trial could be started by pressing the space bar. Every 50 trials, participants were prompted to take a short break.

At the end of the experiments, participants were asked about any strategy use throughout the experiment (“Did you follow a specific strategy throughout the experiment when making your own key choices (i.e., when the ***** were presented)?”; translated from German). Participants had to select one of two response options (“yes”/“no”). If they selected “yes,” a follow-up question required them to specify which strategy they had used.

### Results

Reaction times, error rates and response repetition rates were analyzed using repeated-measures analysis of variance (ANOVA). The R2 response repetition rate was computed as the probability of repeating the prime response R2 as probe response R2 when the prime response R1 also repeated as the probe response R1.

#### Data processing

Data processing and analysis were done with R (R Core Team, 2019; R Version 4.3.0). Ten participants reported having used strategies. Importantly, the result pattern of the binding effect as well as the response repetition effect was identical if these participants were excluded.^3^ For all the analyses, only trials with correct prime response R1 and R2 and correct probe response R1 were included. The rate for at least one error in the prime responses (R1 or R2) was 10.3%. Probe R1 error rate was 5.2% (only including trials without errors in the previous responses). Additionally, due to the logic in the response–response paradigm that a response can never be repeated as immediate subsequent responses but only from prime to probe (Moeller & Frings, 2019), this necessitated the exclusions of trials in which probe response R1 was repeated as probe response R2 in free-choice (8.9% of the trials).^4^

For identifying and excluding responses at the individual participant level, as well as identifying and excluding outliers, the following methods were used. To analyze outliers in RTs, we followed the classification of Tukey (1977, pp. 39–43) and detected outliers using boxplots. In Experiment 1a, no participant had to be excluded due to being an outlier. On the participant’s level, responses were identified and excluded from the analyses as RTs that were more than 1.5 interquartile ranges above the third quartile of the participant’s RT distribution (Tukey, 1977) and RTs below 200 ms were excluded from the analyses. Due to these constraints, 6.9% of the trials were excluded from the RT analyses^5^.

Two analyses of the free-choice data were conducted in the following. For the analysis of RTs, RT in probe R2 was the dependent variable of interest. If the two responses R1 and R2 in the prime were integrated, repeating prime response R1 as probe response R1 should trigger retrieval of the second prime response R2, thus influencing probe R2 RT. See Table 1 for mean RTs.

**Table 1:** Mean reaction times (in ms) for probe response R2 in Experiment 1a, as a function of R1 relation from prime to probe and R2 relation from prime to probe

	R2 relation	
R1 relation	R2 repetition	R2 change
R1 repetition	492	504
R1 change	520	481

For the analysis of response repetitions (e.g., Hommel, 2007; Moeller et al., 2019), our dependent variable of interest was the rate at which participants repeated their R2 response from prime to probe. If the two responses R1 and R2 in the prime were integrated, repeating prime response R1 as probe response R1 should lead to more probe response R2 repetitions than if prime response R1 changed to probe response R1.

#### Reaction times

**Fig. 2:**
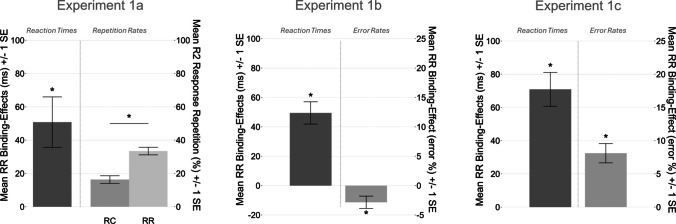
Response–response binding effects and response R2-repetition rates in Experiment 1a, Experiment 1b, and Experiment 1c. *Note*. Response–response binding effects in reaction times and response R2-repetition rates as a function of R1 relation from prime to probe in Experiment 1a, in which probe response R2 was a free-choice response. Response–response binding effects in reaction times and error rates in Experiment 1b, in which probe response R1 was a free-choice response. Response–response binding effects in reaction times and error rates in Experiment 1c, in which prime response R2 was a free-choice response. The response–response binding effect was computed as the advantage of probe R1 repetition over probe R1 change in probe R2 repetition trials minus the advantage of probe R1 repetition over probe R1 change in probe R2 change trials ([R1cR2r-R1rR2r]−[R1cR2c-R1rR2c]). *p < .05 indicates that these individually calculated binding effects were significantly larger than zero. Error bars indicate within-participants standard error of the mean

A 2 (R1 relation: repetition vs. change) × 2 (R2 relation: repetition vs. change) ANOVA on probe R2 RTs yielded a significant main effect of R2 relation, *F*(1,53) = 5.55, *p* = .022, ${{\upeta }_{P}}^{2}$ = .09. Participants responded faster if response R2 changed from prime to probe (*M* = 492 ms, *SD* = 121 ms) than if it repeated (*M* = 506 ms, *SD* = 138 ms). Importantly, the interaction between R1 relation and R2 relation was significant,* F*(1, 53) = 11.20, *p* = .002, ${{\eta }_{P}}^{2}$ = .17, indicating a response–response binding effect (see Fig. 2). The main effect of R1 relation was not significant, *F* < 1, *p* > .5, η^2^_*p*_ < .01.

#### Response repetitions

An ANOVA with the factor R1 relation (repetition vs. change) on probe response R2 repetitions yielded a significant main effect of R1 relation, *F*(1, 53) = 28.08, *p* < .001, ${{\eta }_{P}}^{2}$ = .35. Notably, R2 repetition rates were higher when R1 repeated from prime to probe (*M* = 33.44%, *SD* = 21.7%) than if it changed (*M* = 16.33%, *SD* = 10.1%, see Fig. 2).

### Discussion

In Experiment 1a, we found strong evidence for response–response binding effects in a free-choice scenario. Repeating the first of two individually planned and executed responses retrieves the second response and affects the current action, even if the current action is a free-choice response. We also found similar evidence in probe response R2 repetition rates. That is, participants repeated their probe response R2 in free-choice more often after response R1 repetition as opposed to a R1 response change. The findings demonstrate that the response–response binding effects observed in RTs of a forced-choice task can be replicated in the RTs as well as in response R2 repetition rates.

## Experiment 1b

In Experiment 1b, we investigated whether response–response binding effects occur when the retrieving response is a free-choice response. Therefore, in Experiment 1b, probe response R1 was a free-choice response.

### Method

#### Participants

Sixty-nine participants of Trier University participated in the experiment. As in Experiment 1a, we excluded 14 participants that repeated the prime R1 response as probe R1 response in more than 90% or less than 10% of the trials. The data of one participant were excluded from the analyses because the person was an outlier in error rates (more than 12.5% see Tukey, 1977). The final sample consisted of 54 participants (38 women, 15 men, one person did not wish to disclose; 52 right-handers) with a median age of 22 years (range: 18–32 years). As in Experiment 1a this sample size was sufficient to find a basic response–response binding effect (statistically as the two-way interaction of the factors R1 relation and R2 relation; Moeller & Frings, 2019) of ${{\upeta }_{P}}^{2}$ = 0.20, with alpha = .05 (two-tailed) and a power of 1 − ß = .95 (Faul et al., 2007).

Participants were recruited via Trier University’s participant platform (Sona Systems; sona-systems.com) and performed the experiment online on the experimental platform Pavlovia (Peirce & MacAskill, 2018). All participants consented via online form before participating and received course credit as compensation. As Experiment 1a, this study was done in accordance with the ethics guidelines declared by the ethics committee of Trier University.

#### Design

The design was adjusted from Experiment 1a and comprised two within-subjects factors—namely, response R1 relation from prime to probe (response repetition vs. response change) and response R2 relation from prime to probe (response repetition vs. response change).

#### Apparatus and stimulus

Apparatus and stimuli were the same as in Experiment 1a.

#### Procedure

The procedure was almost identical to that in Experiment 1a, with the following difference: probe response R1 was a free-choice response, while the other responses in the trial were forced-choice responses (see Fig. 1). Thus, the factor response R1 relation from prime to probe was determined by the free-choice of probe response R1 (repetition vs. change). The factor response relation R2 between prime and probe (repetition vs. change) was varied orthogonally, resulting in 200 of each of the two conditions R2 repetition and R2 change.

### Results

Data processing for Experiment 1b was the same as for Experiment 1a. Four participants reported having used strategies. The result pattern was identical if these participants were excluded.^6^ As in Experiment 1, performance in probe R2 was the dependent variable of interest. For the analysis of RTs, only trials with correct prime response R1, prime response R2 and probe response R2 were included. The rate for at least one error in the prime responses (R1 or R2) was 10.3%. Probe R2 error rate was 4.8% (only including trials without errors in the previous responses). Additionally, trials in which the prime response R2 was repeated as probe response R1 in free-choice (21.7% of the trials) and trials in which the probe response R1 was repeated as probe response R2 (25.8% of the trials) had to be excluded.

No outliers in RTs or error rates had to be excluded (Tukey, 1977). On the participant’s level, RTs that were more than 1.5 interquartile ranges above the third quartile of the participant’s RT distribution (Tukey, 1977) and RTs below 200 ms were excluded from the analyses. Due to the exclusion of these responses, 5.2% of the trials were excluded from the RT analyses^7^.

For the analysis of RTs, performance in probe R2 was the dependent variable of interest. If the two responses R1 and R2 in the prime were integrated, repeating prime R1 as probe R1 in free-choice should trigger retrieval of the second prime response R2, thus influencing probe R2 performance. See Table 2 for mean RTs and error rates.

**Table 2:** Mean reaction times (in ms) and mean error rates (in percentages) for probe responses R2 in Experiment 1b, as a function of R1 relation from prime to probe and R2 relation from prime to probe

R2 relation		
R1 relation	R2 repetition	R2 change
R1 repetition	625 (3.3)	638 (3.3)
R1 change	647 (6.9)	610 (6.9)

#### Reaction times

A 2 (R1 relation: repetition vs. change) × 2 (R2 relation: repetition vs. change) ANOVA on probe R2 RTs yielded a significant main effect of R2 relation, *F*(1,53) = 10.33, *p* = .002, ${{\upeta }_{P}}^{2}$ = .16. Participants responded faster if response R2 changed from prime to probe (*M* = 624 ms, *SD* = 96 ms) than if it repeated (*M* = 636 ms, *SD* = 104 ms). Importantly, the interaction between R1 relation and R2 relation was significant,* F*(1,53) = 42.40, *p* < .001, ${{\upeta }_{P}}^{2}$ = .44, indicating a response–response binding effect (see Fig. 2). The main effect of R1 relation was not significant, *F* < 1, *p* > .3, η^2^_*p*_ < .02.

#### Error rates

In the same analysis on error rates, the main effect of R1 relation, *F*(1,53) = 12.05, *p* = .001, ${{\upeta }_{P}}^{2}$ = .19, was significant. Participants made more errors if response R1 changed from prime to probe (*M* = 5.5%, *SD* = 6.0%) than if it repeated (*M* = 3.3%, *SD* = 4.2%). The main effect of R2 relation, *F*(1,53) = 8.75, *p* = .004, ${{\upeta }_{P}}^{2}$ = .14, was significant. Participants made more errors if response R2 changed from prime to probe (*M* = 5.1%, *SD* = 5.6%) than if it repeated (*M* = 3.7%, *SD* = 4.3%). Importantly, the interaction between R1 relation and R2 relation was significant,* F*(1,53) = 7.36, *p* = .008, ${{\upeta }_{P}}^{2}$ = .12, indicating a response–response binding effect (see Fig. 2).

### Discussion

In Experiment 1b, we found response–response binding effects when the retrieving probe response R1 was a free-choice response. This result pattern indicates that response–response bindings appear to be a crucial and robust mechanism in coordinating complex actions, even in scenarios in which the retrieval process is initiated by a freely chosen action.

## Experiment 1c

In Experiment 1c, we investigated whether response–response binding effects occur when the to-be-bound response is a free-choice response. Therefore, in Experiment 1c, prime response R2 was a free-choice response.

### Method

#### Participants

Fifty-four participants of Trier University participated in the experiment (44 women, 12 men; 50 right-handers; median age of 21 years, range: 18–34 years). As in Experiment 1a and 1b this sample size was sufficient to find a basic response–response binding effect (statistically as the two-way interaction of the factors R1 relation and R2 relation; Moeller & Frings, 2019) of ${{\upeta }_{P}}^{2}$ = 0.20, with alpha = .05 (two tailed) and a power of 1 −ß = .95 (Faul et al., 2007).

Participants were recruited via Trier University’s participant platform (Sona Systems; sona-systems.com) and performed the experiment online on the experimental platform Pavlovia (Peirce & MacAskill, 2018). All participants consented via online form before participating and received course credit as compensation. As Experiment 1a, this study was done in accordance with the ethics guidelines declared by the ethics committee of Trier University.

#### Design

The design was adjusted from Experiment 1a and Experiment 1b and comprised two within-subjects factors, namely response R1 relation from prime to probe (response repetition vs. response change) and response R2 relation from prime to probe (response repetition vs. response change).

#### Apparatus and stimulus

Apparatus and stimuli were the same as in Experiment 1a and Experiment 1b.

#### Procedure

The procedure was almost identical to that in Experiment 1a and Experiment 1b, with the following difference: prime response R2 was a free-choice response, while the other responses in the trial were forced-choice responses (see Fig. 1). The factor response relation R1 between prime and probe (repetition vs. change) was varied orthogonally, resulting in 200 of each of the two conditions R2 repetition and R2 change. The factor response R1 relation from prime to probe was varied orthogonally as well, resulting in 200 of each of the two conditions R2 repetition and R2 change.

### Results

Data processing for Experiment 1c was the same as for Experiment 1a and Experiment 1b. No participants had to be excluded due to repetition biases.^8^ Eight participants reported having used strategies. The result pattern was identical if these participants were excluded.^9^ As in Experiment 1b, performance in probe R2 was the dependent variable of interest. For the analysis of RTs, only trials with correct prime response R1, probe response R1 and probe response R2 were included. The rate for errors in prime responses R1 was 6.5%. Probe R1 error rate was 4.5% and probe R2 error rate was 6.8% (only including trials without errors in the previous responses). Additionally, trials with prime response R1 repetition as free-choice prime response R2 were excluded (7.5% of the trials). No outliers in RTs or error rates had to be excluded (Tukey, 1977). On the participant’s level, RTs that were more than 1.5 interquartile ranges above the third quartile of the participant’s RT distribution (Tukey, 1977) and RTs below 200 ms were excluded from the analyses. Due to the exclusion of these responses, 8.1% of the trials were excluded from the RT analyses^10^.

For the analysis of RTs, performance in probe R2 was the dependent variable of interest. If the two responses R1 and R2 in the prime were integrated, repeating prime R1 as probe R1 should trigger retrieval of the second prime response R2, thus influencing probe R2 performance. See Table 3 for mean RTs and error rates.

**Table 3:** Mean reaction times (in ms) and mean error rates (in percentages) for probe responses R2 in Experiment 1c, as a function of R1 relation from prime to probe and R2 relation from prime to probe

R2 relation		
R1 relation	R2 repetition	R2 change
R1 repetition	597 (6.0)	617 (6.8)
R1 change	631 (10.7)	581 (3.3)

#### Reaction times

A 2 (R1 relation: repetition vs. change) × 2 (R2 relation: repetition vs. change) ANOVA on probe R2 RTs yielded a significant main effect of R2 relation, *F*(1,55) = 10.95, *p* = .002, ${{\upeta }_{P}}^{2}$ = .17. Participants responded faster if response R2 changed from prime to probe (*M* = 599 ms, *SD* = 102 ms) than if it repeated (*M* = 614 ms, *SD* = 98 ms). Importantly, the interaction between R1 relation and R2 relation was significant, *F*(1,55) = 48.53, *p* < .001, ${{\upeta }_{P}}^{2}$ = .47, indicating a response–response binding effect (see Fig. 2). The main effect of R1 was not significant, *F* < 1, *p* > .8, η_p_^2^ < .01.

#### Error rates

In the same analysis on error rates, the main effect of R2 relation, *F*(1, 55) = 28.67, *p* < .001, ${{\upeta }_{P}}^{2}$ = .34, was significant. Participants made less errors if response R2 changed from prime to probe (*M* = 5.0%, *SD* = 6.6%) than if it repeated (*M* = 8.3%, *SD* = 6.7%). Importantly, the interaction between R1 relation and R2 relation was significant, *F*(1, 55) = 31.12, *p* < .001, ${{\upeta }_{P}}^{2}$ = .36, indicating a response–response binding effect (see Fig. 2). The main effect of R1 was not significant, *F* < 1, *p* > .3, η_p_^2^ < .02.

### Discussion

In Experiment 1c, we found response–response binding effects when the to-be-bound prime response R2 was a free-choice response. This finding suggests that free-choice responses can be bound to forced-choice responses and integrated in higher-order representations of action sequences.

## General discussion

In the present study, we investigated whether free-choice response features can be bound and retrieved within action sequences, thereby providing action control short-cuts previously only demonstrated for forced-choice actions. For this purpose, we used a response–response binding task consisting of sequences of four individually executed responses. In three experiments, participants selected either the to-be-bound response (prime R2), the retrieving response (probe R1), or the current response (probe R2) endogenously in free-choice, while the other responses in the sequence were selected exogenously in forced choice. As hypothesized, Experiment 1a demonstrated that binding and retrieval of forced-choice responses influenced performance in a current free-choice action, as indicated in both response–response binding effects as well as response repetitions effects in reaction times. Experiment 1b provided evidence suggesting that a free-choice response can initiate the process of retrieval of previously bound forced-choice responses, as indicated in response–response binding effects. Finally, Experiment 1c showed that a free-choice response can itself be bound to a forced-choice response and be later retrieved, as reflected in response–response binding effects.

Beyond these general findings, some more specific results merit further discussion. First, according to the BRAC framework, probe R1 repetitions should trigger retrieval of the bound prime R2, thereby facilitating probe R2 repetitions and impairing changes, whereas no such retrieval should occur for probe R1 changes. Yet even though probe R1 cannot trigger retrieval in R1 change trials, the same R2 is still bound to a different preceding response. Hence, executing this response in direct succession of an unrelated response is impaired compared with the execution of another unrelated response. Thus, our descriptive pattern of stronger performance differences in cases of probe R1 changes can be explained within this framework, as these trials involve additional interference from bindings with unrelated preceding responses. Furthermore, Experiment 1b revealed a negative response–response binding effect in the error rates, which stood in contrast to the positive binding effect observed in reaction time data. Importantly, negative binding effects were previously observed in forced-choice paradigms and were discussed to depend on attentional mechanisms (e.g., nonspatial inhibition of return; Schöpper et al., 2022). Thus, this pattern is not specific to free-choice tasks and may reflect attentional influences or task-specific noise. However, future studies are needed to clarify the cognitive underpinnings of such negative binding effects and their implications for models of action control. Finally, Experiment 1b showed a higher exclusion rate of trials due to response repetitions in subsequent responses (21.7%) compared with Experiment 1a (8.9%) and Experiment 1c (7.5%). A likely explanation for this is the position of the free-choice response within the sequence: in Experiment 1b, the free-choice response occurred as probe R1 after a response–stimulus interval following prime R2. This temporal separation may have segmented the action sequence into separate events, such that prime R2 and probe R1 were perceived as less related than the responses within the prime in Experiment 1c or within the probe in Experiment 1a (for the influence of event segmentation on response–response bindings, see Moeller et al., 2025). Consequently, participants may have been more likely to repeat the same key from prime response R2 to probe response R1, compared to repeating responses within prime (Experiment 1c) or probe (Experiment 1a).

Extending prior research, our results not only suggest that free-choice and forced-choice actions are governed by common representational mechanisms (i.e., are bound/integrated into the same event file; see also Richardson et al., 2020), but also that they can be retrieved from these representations and even initiate the retrieval of features from previously bound forced-choice action episodes. These findings challenge dual-system accounts of human action control that assume separate mechanisms for endogenous and exogenous selection (e.g., Herwig et al., 2007; Herwig & Waszak, 2009, 2012; Waszak et al., 2005). Instead, they align with a growing body of evidence suggesting that both types of actions are controlled and monitored through the same processes (Janczyk, Dambacher, et al., 2015a; Janczyk et al., 2012; Janczyk, Nolden, et al., 2015b; Pfeuffer et al., 2023; Pfister et al., 2011). Ultimately, they support the ideomotor assumption that both endogenous and exogenous actions are represented in terms of their anticipated action effects, and any anticipation of the action effect, whether through endogenous or exogenous action selection, leads to response activation (Hommel & Wiers, 2017).

Assuming feature integration and retrieval as a flexible system that can be adaptively operated (Frings et al., 2020), one might also expect that in free-choice situations, the response chosen would be the one offering the greatest benefit for the current action. In fact, in the present study we did find such a response repetition effect. Notably, while previous studies on free-choice responses in stimulus–response binding typically involved participants selecting one of two response alternatives (e.g., Hommel, 2007; Moeller et al., 2019), our findings demonstrate that this response repetition effect persists even when participants are required to select from four response alternatives. Such a response repetition effect as result of choosing the least demanding response has been also suggested as a bias to conserve cognitive effort (e.g., Richardson et al., 2020). These results emphasize the role of binding and retrieval processes in facilitating future behavior based on past experienced sensorimotor experiences. Importantly, this facilitation seems to be evident not only in how efficiently a response is performed (e.g., through reduced reaction times and error rates) but also in the preference for specific responses when multiple options are available. These findings highlight binding and retrieval mechanisms as fundamental and highly flexible mechanisms in action control that can shape both the performance and selection of actions in complex action sequences involving different modes of action selection (Frings et al., 2020).

It should be noted that previous studies have often avoided using RTs as the dependent variable in free-choice tasks due to the concern that RTs might be overly influenced by explicit strategies (Hommel, 2007; but see Dutzi & Hommel, 2009; Herwig & Waszak, 2012; Moeller et al., 2019, for analyses of free-choice effects in RTs). In fact, it is reasonable to assume that when choosing between two actions, resulting in either a repetition or a change of the previously executed action, participants are more likely to employ explicit strategies, such as alternation or repetition strategies. In contrast, our response–response binding task required participants to choose between four action options, reducing the probability of explicit strategies influencing response selection (cf. Tempel & Frings, 2017).

With the present studies we investigated binding and retrieval of free-choice actions separately, showing that free-choice responses are not only significantly affected by binding and retrieval processes but can furthermore engage them. In forced-choice actions, humans have to decide how to coordinate and execute their response to a stimulus, which requires the planning and execution of individual movements associated with a single goal. In contrast, free-choice actions require selecting among multiple possible movements and consequently action goals, often under time pressure (cf. Gallivan, 2018). Crucially, our cognitive system seems to rely on the same binding and retrieval processes to select, plan and execute movements regardless of whether one is engaging in forced-choice or free-choice actions. That is, we demonstrated that binding and retrieval of responses influence not only performance but transfer to ‘simple’ decision-making (i.e., decision-making in situations with minimal information and very few alternatives).

Importantly, current influential cognitive theories of decision-making would not have predicted the result pattern we found in our study. These models primarily focus on factors such as the selection of strategies or heuristics from a repertoire, or cues that trigger possible choice options through spreading activation to influence current decision-making (e.g., Gigerenzer & Todd, 1999; Glöckner & Betsch, 2008; Lee & Cummins, 2004). Although the influence of irrelevant perceptual information on decision-making is being discussed in decision-making research (paralleling distractor-response binding in action control literature; see, Nett et al., 2015), decision-making models do not account for the impact of previous response episodes on current response decisions as we found. Our findings, therefore, highlight a point at which the incorporation of action control processes into decision-making models might be sensible. Addressing this gap could provide a more comprehensive understanding of how previous action episodes shape present cognitive and behavioral outcomes in decision-making situations.

A limitation of the present studies (and of most laboratory investigations on free-choice actions) is that the free choice was restricted to four equally plausible response options. Notably, this already presents twice the number of options typically used in free-choice studies on binding, which often relied on two response options. However, the present studies still capture only a simplified version of the complexity in real-world decision-making, where individuals often choose among a much broader set of action alternatives that are plausible in a certain situation. Moreover, the present study was not designed to directly compare binding effects involving free-choice and forced-choice responses. Previous studies using action-effect compatibility paradigms revealed effects of comparable size for both types of actions (e.g., Janczyk, Dambacher, et al., 2025a, Janczyk et al., 2017). To further examine whether free-choice and forced-choice actions result in comparable strong binding effects, future research could employ yoked designs in which the frequency of free-choice responses is matched to those of forced-choice responses. Such designs would allow for a systematic assessment of similarities and differences in action control under endogenous versus exogenous decision conditions.

In sum, the present findings together with studies suggesting that responses do not even have to be executed for binding and retrieval mechanisms to occur (Nemeth et al., 2024) and that binding of the intended response (not the executed one) takes place in the face of an error (Foerster et al., 2022, 2023), emphasize that binding and retrieval fundamentally operate on goal representations (Frings et al., 2020). While the mode of response selection and the actual execution of a response may modulate the strength of these processes, the current findings highlight binding and retrieval as flexible mechanisms of action control, ensuring the coordination of goal-directed behavior across different modes of action selection.

## Conclusion

The ability to have control over choosing one’s own action (and its consequences) is a key aspect of action taking and our role as active agents in the world (Schwarz et al., 2019). In line with the assumption that binding and retrieval mechanisms apply to both exogenous and endogenous actions, we demonstrated here for the first time that free-choice responses are not only bound and retrieved by forced-choice actions, but also able to initiate event file retrieval of previously bound responses in a more complex action sequence. Our results therefore not only suggest that binding and retrieval mechanisms influence current performance but also influence decision-making in situations involving high degrees of agency. Our findings contribute to a growing body of literature suggesting that binding and retrieval processes are highly flexible and fundamental mechanisms in action control.

## Data Availability

The data of the experiments is available at OSF (https://osf.io/t7pjb/?view_only=1af9b6ce0c1c4e19ab7a79a4b0bd176f). None of the experiments was preregistered.

## Appendix 1

Appendix Table 4

**Table 4:** Self-report of participants that indicated having used a strategy in Experiment 1a (translated from German)

Participant	Reported strategy
1	*Rhythm strategy.*
2	*I usually chose the number that was missing in the sequence, or in the case of 3 and 4, if a 3 came up again, I took the 4 again.*
3	*I tried to finish the sequence.*
4	*Yes, but not all the time, just now and then, like, “Now I have to press this or that again.”*
5	*Sometimes I noticed that I automatically followed a sequence (e.g., 1234 or 1414 or 3434), but I tried to follow it sometimes and not other times.*
6	[Not further specified]
7	[Not further specified]
8	[Not further specified]
9	[Not further specified]
10	[Not further specified]

## Appendix 2

Appendix Table 5

**Table 5:** Self-report of participants that indicated having used a strategy in Experiment 1b (translated from German)

Participant	Reported strategy
1	[Not further specified]
2	[Not further specified]
3	[Not further specified]
4	[Not further specified]

## Appendix 3

Appendix Table 6

**Table 6:** Self-report of participants that indicated having used a strategy in Experiment 1c (translated from German)

Participant	Reported strategy
1	*I usually pressed the keys next to me on the star keypad. When C came up and then star, I pressed D, and so on.*
2	*Press the same button again as before.*
3	*I mostly used the following key*
4	*I pressed the key I had pressed before, because the asterisk key always came second.*
5	*Focus fully on the four keys, press the space bar with your right thumb, and breathe in deeply during the pauses.*
6	*I pressed the key that I hadn't pressed for the longest time (based on my intuition).*
7	*The asterisk is always in second place, so in most cases it is the key marked with an exclamation mark.*
8	[Not further specified]

## Footnotes

1 Note that the procedures in Experiment 1b and Experiment 1c only allow for the analysis of RTs and error rates, not response repetitions, since probe R2 response was always in forced choice; whereas Experiment 1a only allows for the analysis of RTs and response repetitions, but not error rates, since probe R2 response was always in free choice (see Fig. 1).

2 Note that in prior studies employing similar free-choice procedures, participants were likewise asked to respond randomly while avoiding systematic key use throughout the entire experiment (e.g., `Hommel, 2007; `Moeller et al., 2019; `Richardson et al., 2020).

3 Analyses excluding participants who reported having strategies: In the 2 (R1 relation: repetition vs. change) × 2 (R2 relation: repetition vs. change) ANOVA on performance in probe R2: In RTs, the main effect of R2 relation was not significant, *F*(1,43) = 2.63, *p* = .122, η_p_^2^ = .06; however, importantly, the interaction of R1 relation and R2 relation was significant, *F*(1,43) = 6.60, *p* = .014, η_p_^2^ = .13. Notably, an effective sample size of 44 participants still ensures a power of 1 − β = .90 for finding an effect of *d* = 0.5, with alpha = .05 (two-tailed). In the ANOVA with the factor R1 relation (repetition vs. change) on probe response R2 repetitions the main effect of R1 relation was not significant,* F*(1, 43) < 1, *p* > .970, ηp^2^ < .01.

4 Note that identifying and excluding participants based on uneven key usage across the experiment (specific key usage of <10% or >90%; Experiment 1a: six participants; Experiment 1b: ten participants; Experiment 1c: 18 participants) did not change the overall pattern of results.

5 Note that the mean (median and *SD* in parentheses) of trials in each R1 relation – R2 relation condition were as follows: RCRC: 121 (123; 33), RCRR: 21 (21; 13); RRRC: 94 (99; 37, RRRR: 46 (34; 34) trials.

6 Analyses excluding participants who reported having strategies: 2 (R1 relation: repetition vs. change) × 2 (R2 relation: repetition vs. change) ANOVA on performance in probe R2: In RTs, the main effect of R2 Relation, *F*(1,49) = 8.50, *p* = .005, η_p_^2^ = .15, and the interaction of R1 relation and R2 relation was significant, *F*(1,49) = 38.07, *p* < .001, η_p_^2^ = .44. In error rates, the main effect of R1 relation was significant, *F*(1,49) = 9.68, *p* = .003, η_p_^2^ = .16. The main effect of R2 relation was significant, *F*(1,49) = 6.77, *p* = .012, η_p_^2^ = .12. The interaction of R1 relation and R2 relation was also significant, *F*(1,49) = 6.50, *p* = .014, η_p_^2^ = .12.

7 Note that the mean (median and *SD* in parentheses) of remaining trials in each R1 relation – R2 relation condition in reaction times in each PPREL condition were as follows: RCRC: 52 (50; 19), RCRR: 99 (96; 38); RRRC: 29 (26; 16), RRRR: 30 (28; 15). The mean (median and *SD* in parentheses) of remaining trials in error rates in each R1 relation – R2 relation condition were as follows: RCRC: 58 (57; 21), RCRR: 108 (105; 21); RRRC: 32 (28;16), RRRR: 33 (31; 16).

8 Note that discrepancy in participant exclusions across Experiments 1a–1c was probably to the position of the free-choice response within the trial sequence. In Experiments 1a and 1b, probe responses were free-choice, which led to extreme repetition biases in some participants and thus required exclusions. In Experiment 1c, by contrast, probe responses were in forced-choice and the distribution of repetitions and changes was experimentally controlled, depending on which free-choice response had been selected at prime R2.

9 Analyses excluding participants who reported having strategies: 2 (R1 relation: repetition vs. change) × 2 (R2 relation: repetition vs. change) ANOVA on performance in probe R2: In RTs, the main effect of R2 relation, *F*(1,46) = 10.17, *p* = .003, η_p_^2^ = .18, and the interaction of R1 relation and R2 relation was significant, *F*(1, 46) = 36.60, *p* < .001, η_p_^2^ = .44. In error rates, the main effect of R2 relation, *F*(1,46) = 20.56, *p* < .001, η_p_^2^ = .31, and the interaction of R1 relation and R2 relation was significant, *F*(1, 46) = 23.55, *p* < .001, η_p_^2^ = .34. Notably, an effective sample size of 46 participants still ensures a power of 1 − β = .91 for finding an effect of *d* = 0.5, with alpha = .05 (two-tailed).

10 Note that the mean (median and *SD*) of remaining trials in reaction times in each PPREL condition were as follows: RCRC: 77 (85; 20), RCRR: 71 (76; 19); RRRC: 74 (80; 20), RRRR: 75 (81; 19). The mean (median and *SD*) of remaining trials in error rates in each PPREL condition were as follows: RCRC: 83 (91; 21), RCRR: 83 (90; 21); RRRC: 82 (88; 20), RRRR: 83 (89; 20).
